# Cerebral Ischemia-Reperfusion Is Associated With Upregulation of Cofilin-1 in the Motor Cortex

**DOI:** 10.3389/fcell.2021.634347

**Published:** 2021-03-11

**Authors:** Ming-Shu Xu, Lei-Miao Yin, Ai-Fang Cheng, Ying-Jie Zhang, Di Zhang, Miao-Miao Tao, Yun-Yi Deng, Lin-Bao Ge, Chun-Lei Shan

**Affiliations:** ^1^Yueyang Hospital of Integrated Traditional Chinese and Western Medicine, Shanghai University of Traditional Chinese Medicine, Shanghai, China; ^2^Shanghai Research Institute of Acupuncture and Meridian, Shanghai University of Traditional Chinese Medicine, Shanghai, China

**Keywords:** cerebral ischemia-reperfusion, gait analysis, cofilin-1, motor cortex, penumbra

## Abstract

Cerebral ischemia is one of the leading causes of death. Reperfusion is a critical stage after thrombolysis or thrombectomy, accompanied by oxidative stress, excitotoxicity, neuroinflammation, and defects in synapse structure. The process is closely related to the dephosphorylation of actin-binding proteins (e.g., cofilin-1) by specific phosphatases. Although studies of the molecular mechanisms of the actin cytoskeleton have been ongoing for decades, limited studies have directly investigated reperfusion-induced reorganization of actin-binding protein, and little is known about the gene expression of actin-binding proteins. The exact mechanism is still uncertain. The motor cortex is very important to save nerve function; therefore, we chose the penumbra to study the relationship between cerebral ischemia-reperfusion and actin-binding protein. After transient middle cerebral artery occlusion (MCAO) and reperfusion, we confirmed reperfusion and motor function deficit by cerebral blood flow and gait analysis. PCR was used to screen the high expression mRNAs in penumbra of the motor cortex. The high expression of cofilin in this region was confirmed by immunohistochemistry (IHC) and Western blot (WB). The change in cofilin-1 expression appears at the same time as gait imbalance, especially maximum variation and left front swing. It is suggested that cofilin-1 may partially affect motor cortex function. This result provides a potential mechanism for understanding cerebral ischemia-reperfusion.

## Introduction

Cerebral ischemia (ischemic stroke) is one of the leading causes of death and disability worldwide (Wang et al., 2018; Virani et al., 2020). Mainly because of the expanding and aging population, the absolute number of related deaths is increasing (Feigin et al., 2014; Vivanco-Hidalgo et al., 2019). According to China stroke statistics, more than 3,010,000 inpatients with stroke were admitted to hospitals during 2018; among them, 81.9% had ischemic stroke (Wang et al., 2020). Unfortunately, therapeutic options for ischemic stroke are limited (Wang et al., 2019). In the last 5 years, reperfusion therapies, either intravenous thrombolysis or mechanical thrombectomy, have been the first line of care in a growing number of eligible acute ischemic stroke patients (Powers et al., 2018; Chamorro et al., 2020). There are potential risks in the application of reperfusion (Huang et al., 2019; Thorén et al., 2020). Reperfusion plays a biphasic role: it is beneficial in the acute stage but is potentially deleterious during recovery. Ischemia-reperfusion may result in reperfusion injury, which manifests as hemorrhagic transformation, brain edema, infarct progression, and neurologic worsening (Choi and Pile-Spellman, 2018). Most of these changes are caused by the energy exhaustion of neurons, followed by Ca^2+^ entry (Stegner et al., 2019), cell edema (Liebeskind et al., 2019) and excitotoxicity (Lai et al., 2014), neuroinflammation (Jayaraj et al., 2019), apoptosis (Chen et al., 2020), and autophagy (Yan et al., 2013; He et al., 2020). While irretrievable neuronal loss occurs after a series of spatiotemporal pathological changes, especially in the infarct core area where blood flow drops quickly, the surrounding hypoperfused penumbra region (peri-infarct area) is at risk of delayed cell death. Neuroprotection aims to preserve the penumbra (Chamorro et al., 2020), especially the penumbra region in the motor cortex. Neuroprotection includes not only reducing injury (such as anti-inflammation and anti-apoptotic), but also promoting restoration (such as neurogenesis and regeneration). Neurogenesis under physiological and pathological conditions was found in hippocampal and motor cortex (Chamaa et al., 2018; Lv et al., 2019; Wu et al., 2020), but different results were reported (Sorrells et al., 2018). MCAO model leads to the injury of cortex, basal ganglia (Longa et al., 1989; Ma et al., 2020), and motor deficit induced by MCAO is partially related to motor cortex. Rodent motor cortex plays an important role in motor control (Peters et al., 2017; Bundy et al., 2019; Veuthey et al., 2020). If the motor cortex injury could be treated in time, motor function may be recovery gradually (Wang et al., 2021). These injuries are related to oxidative stress (Chamorro et al., 2016), excitotoxicity (Lai et al., 2014), and neuroinflammation (Jayaraj et al., 2019) induced by reperfusion. All of these factors will cause changes in actin-binding proteins.

Actin-binding proteins (such as cofilin-1) play an important role in the regulation of skeletal proteins in neurons (Bernstein and Bamburg, 2010; Shirao et al., 2017), in the injury and repair of neurons (Bamburg et al., 2010; Alhadidi et al., 2016; Shah and Rossie, 2018), apoptosis and autophagy (Head et al., 2009; Sanchez-Varo et al., 2012; Madineni et al., 2016; Wang et al., 2016), and in changes in mitochondrial function and structure (Hoffmann et al., 2019). Actin-binding proteins also play an important role in the regeneration and development of dendritic spines in a pathological state (Sekino et al., 2007; Koganezawa et al., 2017; Kreis et al., 2019). Existing studies have shown that these actin-binding proteins are changed in degenerative neuropathy (Shaw and Bamburg, 2017; Schaffer et al., 2018; Inoue et al., 2019). Cofilin-1 is abnormal after ischemia and affects the morphological integrity, receptor transport and signal transduction of spines in synapses (Shaw and Bamburg, 2017; Won et al., 2018; Shu et al., 2019). Arp2/3 is also commonly involved in actin cycling/turnover (Korobova and Svitkina, 2008; Spence et al., 2016; Konietzny et al., 2017). Additionally, it was found that drebrin plays an important role in developing cerebral cortex and neurodegenerative diseases (Chimura et al., 2015; Sinclair et al., 2015; Inoue et al., 2019). The abnormal changes in actin-binding proteins, which are major regulators of actin dynamics, may result in dendritic injury of neurons during ischemic stroke (Shu et al., 2019). The changes in nerve function induced by actin-binding proteins are not the same in different brain regions during cerebral ischemia-reperfusion. Limited studies have focused on actin-binding proteins in the motor cortex, and little is known about the gene expression of these proteins.

At present, it is not clear whether there are changes in these actin-binding proteins during cerebral ischemia-reperfusion in the motor cortex penumbra. In this study, we aimed to identify the regulatory factors (cofilin-1, Arp2/3, and drebrin-like) of actin cycling/turnover related to cerebral ischemia-reperfusion. And the changes in these proteins and mRNAs in the motor cortex penumbra need to be observed. So that we could investigate the pathological processes and understand the effects of actin-binding proteins during cerebral ischemia-reperfusion.

## Materials and Methods

### Grouping and Experimental Process

Experiments were performed in adult male Sprague–Dawley rats (257 ± 19 g, Shanghai SLAC Laboratory Animal Co., Ltd.). Animals were housed in institutional animal facilities on a 12 h day/night cycle with *ad libitum* access to food and water. All experimental procedures were performed in accordance with animal welfare and ethical principles and were approved by the Animal Use and Management Committee of Yueyang Hospital of Integrated Traditional Chinese and Western Medicine, Shanghai University of Traditional Chinese Medicine.

All rats were randomly divided into two groups: the control group (control) and the middle cerebral artery occlusion (MCAO) group. Control group (*n* = 8): no MCAO. MCAO group (*n* = 8): MCAO and reperfusion.

**FIGURE 1:**
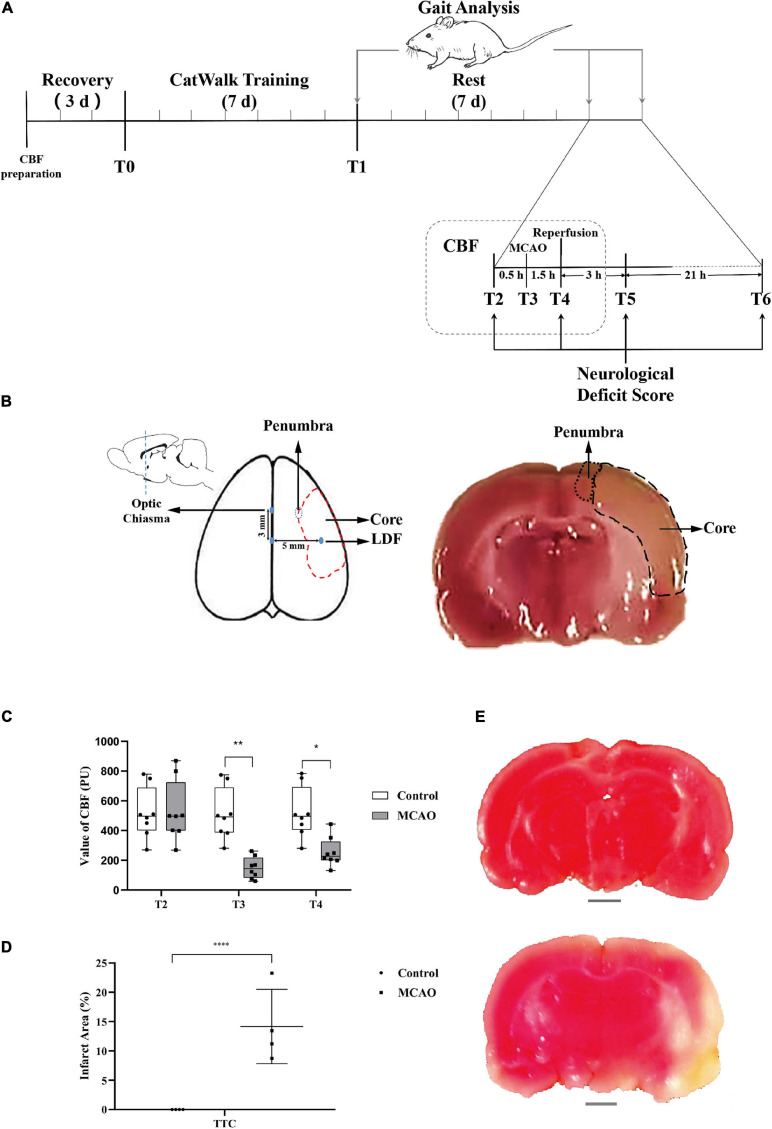
CBF of the motor cortex decreased, and obvious infarction appeared. **(A)** Schematic of experimental design. The experimental rats rested and adapted to the laboratory for 3 days. Gait training was conducted for 7 days starting from T0. Rest occurred for 7 days starting from T1. Middle cerebral artery occlusion was performed at T2. Middle cerebral artery occlusion (MCAO) was successful, and blood flow decreased rapidly at T3. Reperfusion was performed at T4 (1.5 h after middle cerebral artery occlusion). T5 was 3 h after reperfusion. T6 was 24 h after reperfusion, and before anesthesia sacrifice, samples were collected for TTC and molecular biology detection. The time points of gait analysis were T1 (after gait training), T2 (before anesthesia), and T6 (before anesthesia sacrifice). Changes in neurological deficit scores were detected at T2, T4 (after reperfusion recovery), T5 (3 h of reperfusion), and T6 (24 h of reperfusion). CBF was measured at T2, T3 (after middle cerebral artery occlusion), and T4. **(B)** The sketch of the sampling area. The value of CBF was measured with LDF in the area (3 mm posterior to the bregma and 5 mm lateral to the midline). Ischemia core and penumbra were shown with dotted lines (middle and right). A coronal sections through optic chiasm were stained with TTC, and the penumbra was selected for PCR, WB (right). **(C)** Value of CBF. The value of CBF was not significantly different between MCAO rats and control rats at T2, and the value of the MCAO group decreased significantly at T3. Immediately after middle cerebral artery occlusion reperfusion (T4), the CBF value was still significantly lower than that of the control group. Data are presented as the mean ± SD (*n* = 8); *P < 0.05 and **P < 0.01. **(D)** The percentage of infarct area shown by TTC staining. The infarct area (%) represents the percentage of the infarction area (white area) and the whole slice area of coronal section at the optic chiasm. The results of TTC staining showed that the infarct area (%) in the MCAO group was significantly higher than that in the control group. Data are presented as the mean ± SD (*n* = 4); ****P < 0.0001. Scale bar, 0.2 cm. **(E)** Picture showing brain tissue stained with TTC. The above picture shows control brain tissue stained red by TTC, the lower picture shows brain tissue of MCAO rats, and the ischemic area is not dyed red (white area in the picture).

The brief procedure is as follows (also shown in Figure 1A): At the T0 time point, cerebral blood flow (CBF) measurements were prepared (after anesthesia, a small piece of scalp was removed, and detection points were exposed), and the rats then recovered for 3 days, and gait training was conducted for 7 days until the T1 time point with CatWalk^TM^ system XT (Noldus, Wageningen, Netherlands). After that, all rats were analyzed for gait and rested for 7 days. At the T2 time point, gait analysis and neurological deficit scores were measured. CBF was measured after anesthesia in both groups, and surgery was performed in the MCAO group. At the T3 time point, MCAO was performed to block blood flow in the MCAO groups, and the CBF of both groups was measured. At the T4 time point (1.5 h after MCAO), the nylon filament suture was removed, cerebral blood flow was reperfused, CBF was measured again, and the neurological deficit score was measured after the rats regained consciousness. At the T5 time point (3 h after reperfusion), the neurological deficit score was measured. At the T6 time point (24 h after reperfusion), gait and scores were measured.

### Middle Cerebral Artery Occlusion and Reperfusion

The rat model of middle cerebral artery (MCA) ischemia-reperfusion was established according to the literature (Longa et al., 1989; Noh et al., 2020). Rats underwent anesthesia by 4% isoflurane (RWD Life Science, 217180501) with anesthesia machine (R500 RWD Life Science Co., LTD., Shenzhen, China). The right common carotid artery (CCA) and internal carotid artery (ICA) were exposed *via* a midline incision in the neck. In the right external carotid artery (ECA), a suture was used to tie an encased knot at the distal end and a slipknot at the proximal end. The right CCA and ICA were temporarily clamped by a microvascular clip and cut approximately 5 mm from the bifurcation of the right ECA. A nylon filament suture (poly-L-lysine-coated nylon monofilament 0.36 mm, Beijing Cinontech Co., Ltd., Beijing, China) was inserted into the incision of the right ECA. The nylon filament suture was advanced from the right ECA through the CCA and up to the ICA for a distance of 18 ± 0.5 mm to block the origin of the MCA. The right MCA was occluded for 90 min. After that, cerebral blood flow was restored by withdrawal of the nylon thread. The temperature of the animals was maintained at 37 ± 0.5°C through a feedback-adjusted heating blanket (Xu et al., 2013).

### Detection of Cerebral Blood Flow and Pathological Confirmation After Operation

#### Cerebral Blood Flow Measurements

CBF was measured in the right hemisphere of the experimental rats. A moor VMS-LDF^TM^ laser Doppler blood flow monitor (Moor Instruments, Devon, United Kingdom) was used to detect the CBF of rats under inhalation anesthesia. Anesthesia was achieved by face mask-delivered isoflurane (2% induction and 1.5% maintenance in 80% N_2_ and 20% O_2_). After placing the animal in the stereotaxic frame, the dorsal surface of the skull was exposed through a midline cut. A laser Doppler flowmetry (LDF) probe was situated on the skull, 3 mm posterior to the bregma and 5 mm lateral to the midline (Nishimura et al., 2000). The reperfusion was confirmed by CBF measurement after pulling out the suture from ECA.

#### TTC Staining

We determined the infarct volume after 24 h of reperfusion with the 2,3,5-triphenyltetrazolium chloride (TTC) method (Longa et al., 1989). Following neurological function evaluation, four rats in each group were deeply anesthetized by an intraperitoneal dose of 400 mg/kg chloral hydrate and then sacrificed. Each brain was removed and sliced into 2 mm sections using a rodent brain matrix slicer (RBM-4000C; ASI Instruments, Warren, MI, United States) (Xu et al., 2013).

Brain tissue sections were immersed in 10 ml of 2% TTC (Beijing Solarbio Life Science and Technology Co., Ltd., Beijing, China) phosphate buffer solution (pH 8.7) and incubated at 37°C in the dark for approximately 30 min. The brain tissue was turned upside down every 10 min. At this time, the normal brain tissue was dyed dark red, and the cerebral infarction area was not stained (gray white). Then, the brain tissue was immersed in 4% paraformaldehyde phosphate buffer (pH 7.4) and fixed at 4°C for 24 h.

A coronal section through optic chiasm was selected. Areas of red and white staining were measured using an image processing program (ImageJ 1.52P, NIH, United States). The percent of infarction is given by the equation: infarct area (%) = infarct area/total area of slice × 100 (Xu et al., 2013).

The coronal sections through optic chiasm were used for cortex penumbra samples (Nash et al., 2018; Sanchez-Bezanilla et al., 2019). The area was shown in Figure 1B. These samples were used for PCR, immunohistochemistry (IHC), and Western blot (WB).

### Assessment of Behavioral Change and Assessment of Gait Change

#### Neurological Deficit Score

The method that we used in this study for behavioral assessment of focal cerebral ischemia in rats has been described previously. All rats were evaluated by the same trained person. The neurological examination results were scored according to the five point system: zero point, no obvious defect; one point, forelimb flexion; two points, forelimb flexion and lateral push resistance reduction; three points, forelimb flexion, lateral push resistance decreased, unilateral rotation occurred in three consecutive tests; four points, three points symptoms plus decreased consciousness (Yang et al., 2000).

#### Gait Training and Gait Analysis

Before the first recording of the gait, rats were habituated to the laboratory and walkway conditions and trained. Rats were trained for 7 days to cross the runway of the CatWalk^TM^ system, a video-based analysis system, to assess static and dynamic gait parameters. Briefly, the equipment was located in a dark and silent room. Rats were made to travel through a 1.5 m long enclosed glass plate. Animal movement was captured with a camera placed under the walkway and connected to the data acquisition station. A burrow-like house was placed with food at the end of the CatWalk^TM^ runway, which the animals recognized as a safe shelter.

On the last day of training, crossing the CatWalk^TM^ system (three runs per animal) was recorded; these measurements were used as baseline values. Three crossings of the CatWalk^TM^ runway without interruption/hesitation were required for a valid kinematic gait analysis in each animal. Functional studies were performed three times in both groups of rats. A series of behavioral tests was performed before MCAO (T2) and 24 h after reperfusion (T6) (Domin et al., 2016). Gait parameters were labeled right forepaw (RF), right hind paw (RH), left forepaw (LF), and left hind paw (LH). Data were analyzed using CatWalk^TM^ XT 10 software (Fluri et al., 2017).

### Gene and Protein Expression of Actin-Binding Proteins in the Motor Cortex

#### Real-Time PCR and Measurement of mRNA

Real-time PCR was carried out using a real-time PCR system (Light Cycler 480 Instrument II, Roche). The real-time cycler was programmed according to the kit protocol. The threshold cycle number was determined using SDS v1.4 Software, and the reactions were performed in triplicate. Total RNA (5 μg) was reverse transcribed into cDNA by using the RevertAid First Strand cDNA synthesis kit (Invitrogen K1622). For RT-PCR of the cDNA, primer pairs were designed to generate intron-spanning products of 101–150 bp (primer sequences are listed in Table 1). The generation of specific PCR products was confirmed by the melting curve. The expression ratio was calculated according to the formula 2^(Rt–*Et)*^/2^(Rn–*En)*^ as described previously (Livak and Schmittgen, 2001). Transcripts with a twofold increase in expression were considered to be upregulated, and those with a 0.5-fold decrease in expression were considered to be downregulated (Yin et al., 2009).

**TABLE 1:** Primers for real-time PCR (RT-PCR).

**Gene**	**Primer sequence (5′→3′) forward/reverse**
COF1	GACTGCCGCTATGCTCTCTA/CTTGATGGCATCCTTGGAGC
ARPC1A	CGGCTCATCTCTGTCTGCTA/AAAACGTTGTTGGGATGCCA
ARPC2	GTGAACAACCGCATCATCGA/AGGACGCCATCAAAATCTGC
ARPC3	GCGGACAGGACCTTGATCTA/TGGAGTTGCACTTTTGGAGC
ARPC4	ACTTCTCTTCCCAGGTCGTG/ACCCGGACAGAATTGATGGA
ARPC5L	TCACTGGACAGGAATGGCAT/AAGCCTTTTCATGCCACTGG
DBNL	TACCAGAAGACCAATGCCGT/TCTCCTCCTCCTTCTCAGCT
ENAH	ATTCAGAGTGGTGGGCAGAA/TTGCTGCCAAAGTTGAGACC
WASL	GGTGACCATCAAGTTCCAGC/GGCCATCAGACACGGATTTC
BRK1	GCGAGAGATTCACCAGGACT/TCTCACCCTTTGTCACCCTC
GAPDH	TGCCACTCAGAAGACTGTGG/TTCAGCTCTGGGATGACCTT

*COF1, cofilin-1; ARPC1A, actin-related protein 2/3 complex, subunit 1A; ARPC2, actin-related protein 2/3 complex, subunit 2; ARPC3, actin-related protein 2/3 complex, subunit 3; ARPC4, actin-related protein 2/3 complex, subunit 4; ARPC5L, actin-related protein 2/3 complex, subunit 5L; DBNL, developmentally regulated brain like protein; ENAH, enabled homolog; WASL, neural Wiskott–Aldrich syndrome protein; BRK1, Brick1 subunit of SCAR/WAVE actin nucleating complex (Brick1 is also known as hematopoietic stem cell protein 300, HSPC300); GAPDH, glyceraldehyde-3-phosphate dehydrogenase.*

#### Immunohistochemistry and Measurement of Proteins

The target area was embedded in paraffin for standby. The tissue was continuously sliced at a thickness of 3 μM. The tissue was pasted on the slide and baked at 60°C for 1 h to make the tissue adhere tightly to the slide. The slices were dehydrated with 100, 90, 80, and 70% gradient alcohol for 30 min and then washed with tap water for 30 min. The slices were placed into a container containing citric acid hydrochloric acid buffer solution, placed in a high-pressure cooker at 100°C for 6 min, cooled to room temperature, and then removed. H_2_O_2_ solution (3%) was added dropwise, incubated in the dark for 5 min, washed with distilled water for 5 min, and washed with buffer solution three times for 5 min each time. After being sealed with 5% normal goat serum and placed at room temperature for 30 min, the excess liquid was removed. The primary antibody (anti-cofilin-1 primary antibody, ab42824, Abcam Inc., 1:100; anti drebrin primary antibody, ab11068, Abcam Inc., 1:50; Anti-ARPC2 antibody, HPA008352, Sigma Inc., 1:400) was added dropwise, placed overnight in a 4°C incubator, and placed in a 37°C incubator for 30 min. The buffer solution was washed three times for 5 min each time. Fifty microliters of biotin-labeled secondary antibody was added dropwise and incubated at 37°C for 30 min, and the buffer solution was washed three times for 5 min each. Horseradish peroxidase labeled streptase ovalbumin working solution was added and incubated at 37°C for 30 min, and the buffer solution was washed three times for 5 min each. One drop each of reagents A, B, and C was added to the kit in turn to avoid light, mixed well and added to the slices. The color reaction was terminated after 5 min at room temperature. Hematoxylin staining was performed for 3 min. The slices were dehydrated by 70, 80, 90, and 100% gradient alcohol successively, xylene was added to clear the slides for 5–8 min, and then the slices were dried naturally. Neutral gum was added to the slides, and they were covered with a clean coverslip (Han et al., 2009).

#### Western Blot and Measurement of Cofilin

Western blot analysis was used to investigate the cofilin protein expression in rat brain. After being separated by SDS-PAGE, the proteins were electrotransferred onto a PVDF membrane. Then, the membrane was blocked with 5% bovine serum albumin (BSA) for 1 h and analyzed using specific primary antibodies, including anti-cofilin (ab42824, Abcam) and anti-β-actin (4,967, Cell Signaling Technology). Horseradish peroxidase (HRP)-conjugated secondary antibodies were applied, and the membrane was visualized using an electrogenerated chemiluminescence (ECL) detection system.

### Statistical Analysis

The data are presented as the mean ± SD. One-way ANOVA (analysis of variance) followed by the least significant difference (LSD) test or the Games-Howell test (depending on the data and on the hypothesis tested). *Post hoc* analysis was used to analyze the significance of the neurological deficit score, CBF and gait measurement among the two groups. *P* < 0.05 was considered indicative of significance.

## Results

According to the schematic (Figure 1A), modeling, CBF detection, behavioral evaluation, infarct area analysis, and molecular biology of actin-binding proteins in the penumbra were performed in rats at specific time points (T0–T6).

### CBF of the Motor Cortex Decreased, Obvious Infarction Appeared

At T2 before the operation, there was no significant difference in the cerebral blood flow of rats between the groups. At T3, MCAO significantly reduced cerebral blood flow compared to that before ischemia compared with the control group. At T4 (1.5 h after MCAO and the beginning of reperfusion), cerebral blood flow in the MCAO group was still significantly lower than that in the control group. After reperfusion, the cerebral blood flow in the MCAO group recovered significantly, but it was still lower than that before the operation (Figure 1C). TTC staining may reflect the infarct region in the rat brain (Figure 1E). There were significant differences in the infarct area (%) between rats in the control and model groups (*P* < 0.05, Figure 1D).

### Obvious Behavioral Changes During Cerebral Ischemia-Reperfusion

**FIGURE 2:**
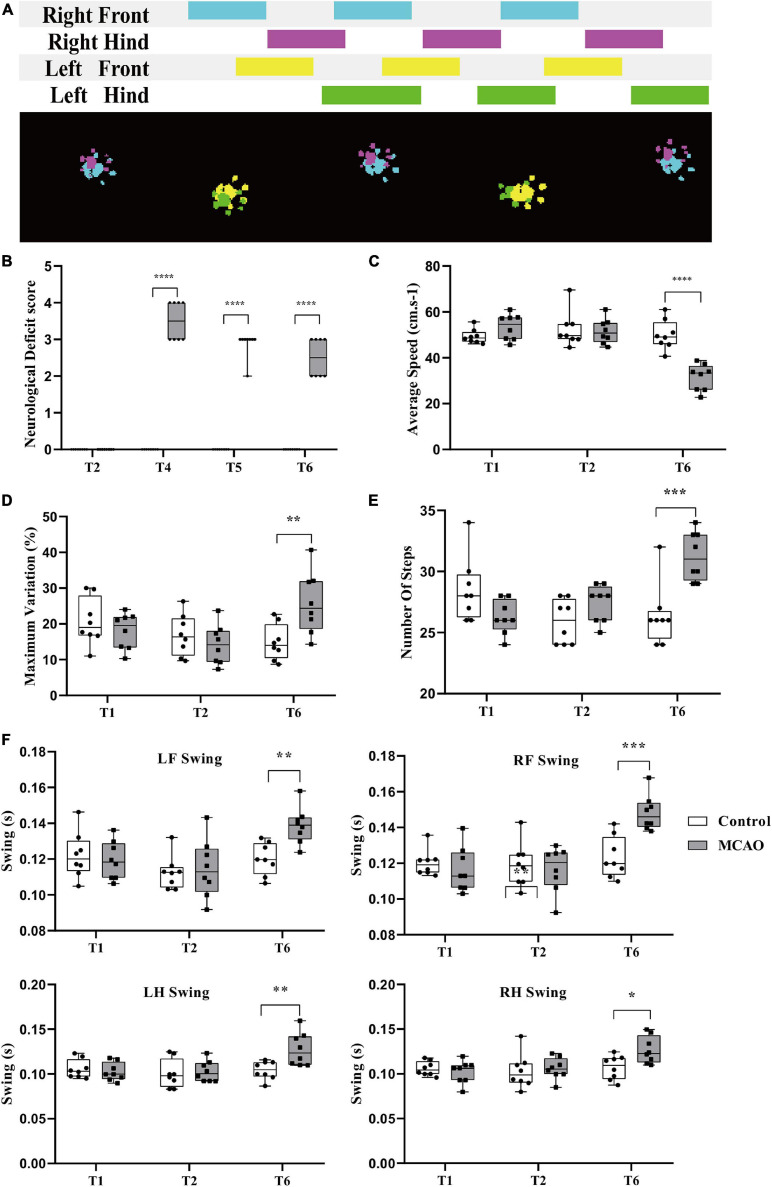
Behavioral changes during cerebral ischemia-reperfusion. **(A)** Acquisition of rat gait parameters by the Catwalk^TM^ system. The rats walked through the runway from left to right. The figure above shows the process of limbs contacting the runway in a time series. The color area is the time of limb contact, and the blank area is the time of limb swing. The figure below shows the limbs in contact with the runway. Right forepaw (RF): blue; right hind paw (RH): fuchsia; left forepaw (LF): yellow; left hind paw (LH): green. **(B)** Neurological deficit score significantly increased. Before middle cerebral artery occlusion (T2), the scores of all groups were 0; immediately after reperfusion (T4), the score of the MCAO group was significantly higher than that of the control group. Compared with the control group, the score in the MCAO group was still significantly higher at 3 h (T5) and 24 h (T6) after reperfusion. **(C,F)** Changes in rat gait during cerebellar ischemia-reperfusion. **(C,D)** The average speed declined, and the maximum variation in the average speed increased. After gait training (T1) and before the middle cerebral artery occlusion operation (T2), there was no significant differences in gait indexes between the control group and the MCAO group. At 24 h after reperfusion (T6), the average speed of the MCAO group was significantly lower than that of the control group. **(E,F)** Number of steps increased and the limb swing expanded. The number of steps was significantly greater than that of the control group. LF swing, RF swing, LH swing, and RH swing were significantly higher than those in the control group. Data are presented as the mean ± SD (*n* = 8); **P < 0.01, ***P < 0.001, and ****P < 0.0001.

To evaluate the change in behavior comprehensively, we used a relatively rough neurologic defect score and relatively reliable gait analysis. Rat gait prints were collected by using the CatWalk^TM^ System. The process of limbs contacting the runway in a temporal series is shown in Figure 2A (upper figure), and the process of the spatial series is shown in Figure 2A (lower figure).

#### Neurological Deficit Score Significantly Increased

The score was determined in conscious rats. At T2, the scores of all rats in the control and MCAO groups were 0. At T4 (beginning reperfusion), the scores of the MCAO group were significantly increased (*P* < 0.05). At T5 (3 h after reperfusion), the score of MCAO rats recovered. At T6 (24 h after reperfusion), the score of MCAO rats was still increased compared with that of control rats, and there was a significant difference (*P* < 0.05) (Figure 2B).

#### Average Speed Decreased, Variation in Average Speed Increased

There was no significant difference in average speed between T1 and T2 and no significant difference in rats between the control group and MCAO group at T1 and T2 (Figure 2C). At T6 (24 h after reperfusion), MCAO decreased the average speed significantly compared with the control group and compared with itself at T1 and T2 (Figure 2C).

The maximum variation is the maximum variation in the average speed of the selected steps in the trial. It can reflect the speed of speed change. After training, control rats are usually able to cross the runway at an approach uniform velocity. Maximum variation is generally 10–20% (Figure 2D). There was no significant difference in maximum variation between T1 and T2. There was no significant difference between groups. At T6 (24 h after reperfusion), the maximum variation increased to some extent, and MCAO led to walking speed that was sometimes fast and sometimes slow. MCAO increased the maximum variation significantly compared with T2. There was a significant difference in maximum variation compared with the control group at T6 (Figure 2D).

#### Number of Steps Increased, Limb Swing Expanded

The number of steps is the total number of selected steps in the run. Limb swing is the duration in seconds of no contact of the paw with the glass plate. After cerebral ischemia-reperfusion, the balance of rats was poor, walking speed was decreased, number of steps increased, and limb swing expanded. There was no significant difference in the number of steps between T1 and T2, and there was no significant difference in the number of steps between the control group and MCAO group at T1 and T2 (Figure 2E). At T6 (24 h after reperfusion), MCAO increased the number of steps significantly compared with the control group and compared with T1 and T2 (*P* < 0.05) (Figure 2E).

The left forelimb was the affected side of the MCAO model. There was no significant difference in LF swing between T1 and T2. There was no significant difference between the groups (Figure 2F). At T6 (24 h after reperfusion), MCAO significantly increased LF swing compared with the control group and compared with T1 and T2 (Figure 2F). The results for RF swing (Figure 2F), LH swing (Figure 2F), and RH swing (Figure 2F) were the same as those for RF swing, and MCAO significantly increased swing compared with the control group.

### Overexpression of COF1 mRNA and COF1 in the Motor Cortex After Cerebral Ischemia-Reperfusion

**FIGURE 3:**
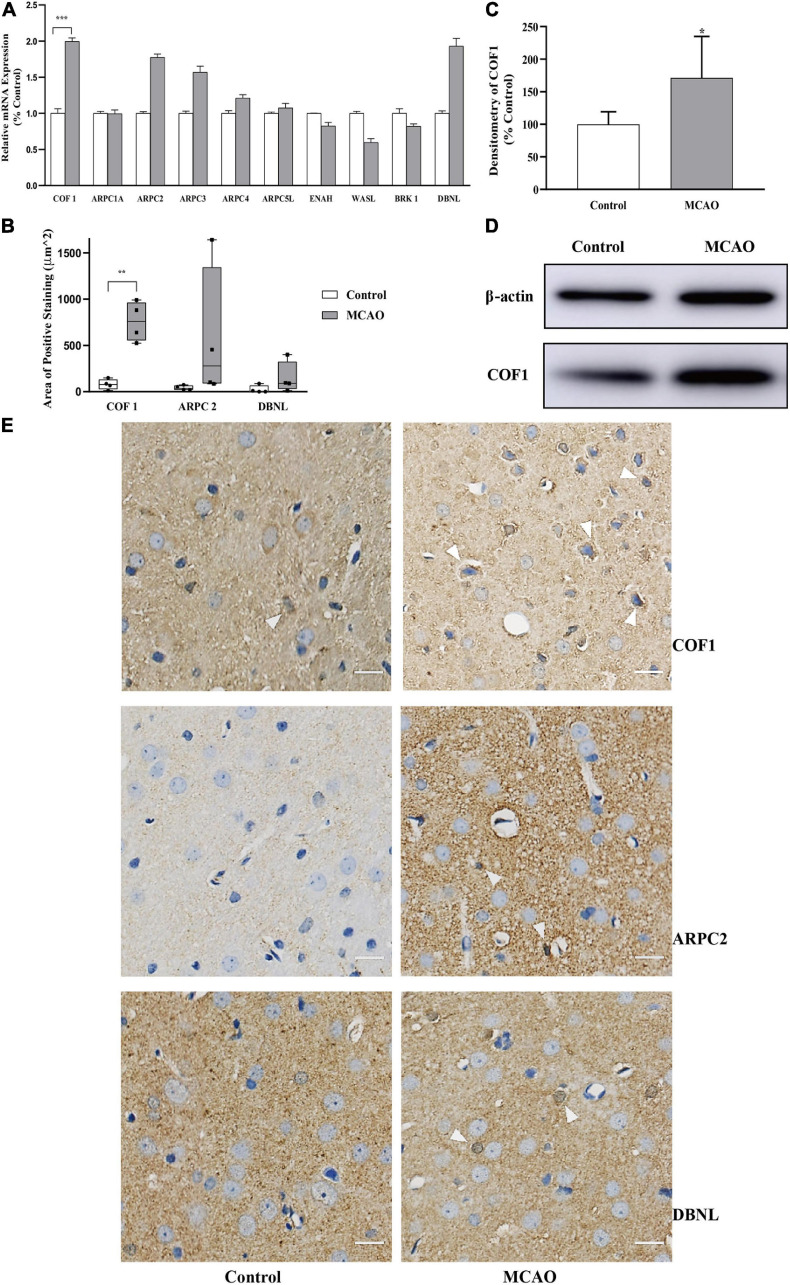
Expression of COF1 mRNA and COF1 after cerebral ischemia-reperfusion **(A)** Relative mRNA expression of actin-binding proteins. The relative mRNA expression of COF1 in the MCAO group was significantly higher than that in the control group. The relative mRNA expression levels of ARPC2, ARPC3, ARPC4, ARPC5L, and DBNL were upregulated less than twofold compared with the control group. The mRNA expression levels of ENAH, WASL, and BRK1 were downregulated less than 0.5-fold compared with the control group. Data are presented as the mean ± SD (*n* = 3); *indicates that the expression levels were upregulated more than twofold compared with the control group. **(B,E)** Area of positive expression COF1, ARPC2, and DBNL. Tissue sections obtained from control and MCAO rats. Sections of MCAO and control tissues were fixed, paraffin embedded, and stained with immunohistochemistry. The nuclei were stained blue with hematoxylin. Immunohistochemistry results: positive neurons exhibited brown cytoplasmic staining (white arrow). The positive expression area of COF1 was significantly increased compared with the control group (COF1). Data are presented as the mean ± SD (*n* = 4); **P < 0.01. Scale bar, 20 μm. **(C,D)** Expression of COF1 protein concentration. After sample preconditioning, the proteins were separated by SDS-PAGE, the COF1 was electrotransferred onto a PVDF membrane. After primary antibody of COF1 and HRP were applied, the COF1 visualized using an electrogenerated chemiluminescence (ECL). The densitometry of COF1 (normalized by β-actin) increased significantly after MCAO and reperfusion. Data are presented as the mean ± SD (*n* = 6); *P < 0.05.

Compared with the control group, the mRNA levels of COF1 were significantly increased by MCAO and reperfusion (Figure 3A). In addition, the mRNA expression levels of ARPC2, ARPC3, ARPC4, ARPC5L, and DBNL were upregulated less than twofold compared with the control group. The mRNA expression levels of ENAH, WASL, and BRK1 were downregulated less than 0.5-fold compared with the control group.

To further verify the mRNA results, three mRNAs (COF1, ARPC2, and DBNL) demonstrating closest to twofold change were selected to verify the protein expression in IHC. For cofilin-1, which is closely related to the polymerization and depolymerization of actin and has obvious changes in this study (Figure 3A), we conducted a semiquantitative analysis of the protein expression using IHC (Figures 3B,E).

The results showed that the positive expression area of COF1 increased significantly after cerebral ischemia-reperfusion (*P* < 0.05) (Figures 3B,E). The positive expression areas of ARPC2 and DBNL did not significantly increase after cerebral ischemia-reperfusion (Figures 3B,E).

To further verify the IHC results, COF1 were selected to verify the protein expression in WB. The results showed that the optical density ratio (normalized by β-actin) of COF1 increased significantly after cerebral ischemia-reperfusion (*P* < 0.05) (Figures 3C,D).

## Discussion

Research on chemical drugs for cerebral ischemia has been conducted for decades. However, currently, the most effective drugs are anticoagulants and thrombolytics, which require a tightly controlled time window (Kepplinger et al., 2016). If used beyond the time window, these drugs will increase the risk of intracranial hemorrhage (Choi and Pile-Spellman, 2018). Vascular recanalization often results in reperfusion injury. Although other drugs (anti-injury or promoting neuron regeneration) have achieved good results in animal experiments, they have not been clinically verified (Kaur et al., 2013; Zhou et al., 2018). These biological processes are closely related to actin-binding proteins (e.g., cofilin-1) through dephosphorylation by specific phosphatases (Alhadidi et al., 2016). Although studies of the molecular mechanisms of the actin cytoskeleton have been ongoing for decades, limited literature has directly investigated cerebral ischemia-reperfusion-induced reorganization of actin-binding proteins in the cortex penumbra, and little is known about the gene expression of actin-binding proteins. Our research explored whether there are changes in actin-binding proteins induced by cerebral ischemic reperfusion.

Some studies have shown that cerebral blood flow decreased nearly 80% after MCAO, and obvious infarct size was found with TTC staining (Beard et al., 2020). Using spontaneously hypertensive rats (Cipolla et al., 2018) and aged hypertensive rats (Chan et al., 2018), similar results were found. Cerebral blood flow and TTC staining results were stronger predictors of brain damage in MCAO rats. Our results are consistent with previous publications.

Sports injuries caused by cerebral ischemia are still the main cause of disability. Upper motor impairment is the most common source of disability after cerebral ischemia (Langhorne et al., 2009; Lin et al., 2019). Similar to human, cerebral ischemia also changed gait in rats. At present, there are limited studies on MCAO using gait analysis. In the past, most studies have focused on static parameters of gait (print area, maximal contact area, and stride length) (Domin et al., 2016; Fan et al., 2018; Hu et al., 2019). Few studies have focused on dynamic parameters (Gao et al., 2020). Our research highlights whole-body dynamic parameters (average speed, maximum variation, number of steps, swing) in gait analysis.

With the help of gait analysis, we can quickly and sensitively evaluate sports injuries and supplement the neurological deficit score. After MCAO on the right brain of the rat, the left limb (especially the left forelimb) has dyskinesia. The left forelimb flexion and extension movement is unfavorable. The movement continuity is poor, and the movement speed is sometimes fast and sometimes slow. Our results partly verified previous study (Parkkinen et al., 2013).

The sports injury (i.e., gait changes) caused by cerebral ischemia is closely related to necrosis of the ischemic area, and the necrotic area will not be repaired (Boned et al., 2017; Baron, 2018). However, the ischemic penumbra belongs to the boundary area. Penumbra may recover after proper treatment or may be aggravated and necrotic (Lo, 2008; Candelario-Jalil and Paul, 2020). Therefore, it is of great value to study the penumbra.

The cytoskeleton has attracted our attention in studying penumbra. It has unique function in forming the major neuron structure. It is essential for protein localization and the transport of molecules between dendrites and axons (Madineni et al., 2016). A large number of studies have confirmed that neural structural reorganization is the basis of functional plasticity (Yamada and Kuba, 2016; Gipson and Olive, 2017). Structural reorganization (e.g., spine) is closely related to skeletal proteins (especially actins). Crosstalk between actin and actin-binding proteins during neural pathology has recently attracted much attention (Carlisle and Kennedy, 2005; Alvarez and Sabatini, 2007; Mar et al., 2014; Tonnesen and Nagerl, 2016; Blanquie and Bradke, 2018). In addition, cerebral ischemia-reperfusion can lead to neural injury. Changes in actin-binding proteins would be shown (Lai et al., 2014; Chamorro et al., 2016; Jayaraj et al., 2019). The function of specific brain regions (such as the motor cortex) would be affected. The study of actin-binding proteins in the penumbra of the motor cortex is helpful to reveal the pathological processes of cerebral ischemia-reperfusion.

Recent studies have emphasized that cofilin-1 is involved in the harmful neuronal processes of ischemic stroke, especially in the penumbra (Won et al., 2018; Shu et al., 2019). Interestingly, cofilin-actin rods were not visible after 1 h, but were widely formed within 4–24 h after reperfusion (Hoffmann et al., 2019).

The cofilin-actin rods form macromolecular aggregates, and affected neurites interrupt the cytoskeleton. The rods also affect organelle transport and lead to loss of dendritic spines (Bamburg et al., 2010). Simple overexpression of cofilin-1 is sufficient to induce actin rod formation and associated neurite degeneration (Minamide et al., 2000). Ischemia also leads to loss of dendrites and spines in neurons, even with survival of the parent cell body (Tanay et al., 2006). Cofilin-actin rod suppression during the acute phase of ischemic stroke might provide neuroprotection with histological results (Kurisu et al., 2019). It has also been reported that cofilin-1 increases in oxygen glucose deprivation model of ischemia with *in vitro* fetal mouse neurons (Madineni et al., 2016). Our study confirmed that MCAO leads to an increase in COF1 mRNA and COF1 protein in the penumbra of the motor cortex with adult rats. These changes may be partially associated with gait imbalance and may be related to structural reorganization and functional impairment of neurons.

Cofilin-1 is an actin-binding protein normally involved in the dynamic turnover of actin filaments (actin treadmilling) and other cell functions (new functions). Phosphoregulation of cofilin is a downstream target of many transmembrane signaling processes, and it has been linked in rodent models to many different neurodegenerative and neurological disorders (Alhadidi et al., 2017; Shaw and Bamburg, 2017). Phosphorylation by LIM kinase (LIMK) causes the deactivation of cofilin, while dephosphorylation by slingshot protein phosphatase-1L (SSH-1L) or chronophin (CIN). Cofilin with active form promotes the turnover of actin filaments (Shu et al., 2019). Cerebral ischemia-reperfusion induces decline in ATP, shifts the balance of kinase/phosphatase activity toward dephosphocofilin, increases active cofilin, increases ADP-actin, and promotes cofilin oxidation and actin rod formation (Bamburg and Bernstein, 2016). Cerebral ischemia-reperfusion activates SSH-1L and CIN, causing active cofilin-1 to emerge and affecting the functions of cofilin-1 (Kanellos and Frame, 2016).

The regulatory mechanism for cofilin mRNA translation during cerebral ischemia-reperfusion is still not well studied. We first reported that the increase in cofilin-1 was associated with cerebral ischemia-reperfusion in the motor cortex. It is suggested that the increase in cofilin-1 is due to dephosphorylation of p-cofilin-1 and a large number of transcriptional mRNAs. In the nucleus, cofilin-1 is required for RNA polymerase II transcription elongation (Obrdlik and Percipalle, 2011), and the presence of cofilin-1 in the nucleus is consistent with enhanced/faster RNA polymerase II-dependent transcription (Domingues et al., 2020). After cerebral ischemia-reperfusion, a large amount of cofilin-1 accumulates in the cytoplasm, which may enter the nucleus and accelerate the transcription of mRNA (including cofilin-1 mRNA). This may be a potential mechanism for the continuous increase in cofilin-1 and damage to the cytoskeleton.

Although previous studies found that ARP2/3 and DBNL exhibit significant changes after cerebral ischemia, we determined that the mRNA and protein levels of ARP2/3 and DBNL exhibit changes, but they are not significant. It has been suggested that ARP2/3 and DBNL may not be direct or key influencing factors in MCAO- and reperfusion-induced sports injury.

Due to the limitation of such tMCAO in this research, the affected areas include cortex and basic ganglia. Whether the above results are applicable to cortex stroke remains to be further verified.

## Conclusion

Cerebral ischemia-reperfusion leads to the motor cortex injury with the cofilin-1 increase in the penumbra and is partially related to dyskinesia, suggesting that cofilin-1 plays an important role during cerebral ischemia-reperfusion.

## Data Availability Statement

The original contributions presented in the study are included in the article/supplementary material, further inquiries can be directed to the corresponding author/s.

## Ethics Statement

The animal study was reviewed and approved by the Animal Use and Management Committee of Yueyang Hospital of Integrated Traditional Chinese and Western Medicine, Shanghai University of Traditional Chinese Medicine.

## Author Contributions

M-SX and L-MY were responsible for the RT-PCR and Western blot validation and drafting of the manuscript. A-FC, Y-JZ, DZ, and M-MT duplicated the rat middle cerebral artery occlusion model and neurological deficit score. Y-YD contributed to the immunohistochemistry analyses. A-FC, Y-JZ, and M-MT performed the gait training and gait analysis. A-FC and M-MT completed the laser Doppler blood flow and TTC staining. M-SX, L-MY, L-BG, and C-LS contributed to the design of the study and draft of the manuscript. All authors read and approved the final manuscript.

## Conflict of Interest

The authors declare that the research was conducted in the absence of any commercial or financial relationships that could be construed as a potential conflict of interest.
